# Artificial Synapses Based on an Optical/Electrical Biomemristor

**DOI:** 10.3390/nano13233012

**Published:** 2023-11-24

**Authors:** Lu Wang, Shutao Wei, Jiachu Xie, Yuehang Ju, Tianyu Yang, Dianzhong Wen

**Affiliations:** Heilongjiang Provincial Key Laboratory of Micronano Sensitive Devices and Systems, School of Electronic Engineering, Heilongjiang University, Harbin 150080, China

**Keywords:** physical transient, biological materials, graphene quantum dots, OR gate

## Abstract

As artificial synapse devices, memristors have attracted widespread attention in the field of neuromorphic computing. In this paper, Al/polymethyl methacrylate (PMMA)/egg albumen (EA)–graphene quantum dots (GQDs)/PMMA/indium tin oxide (ITO) electrically/optically tunable biomemristors were fabricated using the egg protein as a dielectric layer. The electrons in the GQDs were injected from the quantum dots into the dielectric layer or into the adjacent quantum dots under the excitation of light, and the EA–GQDs dielectric layer formed a pathway composed of GQDs for electronic transmission. The device successfully performed nine brain synaptic functions: excitatory postsynaptic current (EPSC), paired-pulse facilitation (PPF), short-term potentiation (STP), short-term depression (STD), the transition from short-term plasticity to long-term plasticity, spike-timing-dependent plasticity (STDP), spike-rate-dependent plasticity (SRDP), the process of learning, forgetting, and relearning, and Pavlov associative memory under UV light stimulation. The successful simulation of the synaptic behavior of this device provides the possibility for biomaterials to realize neuromorphic computing.

## 1. Introduction

Different from the traditional von Neumann computer architecture, neuromorphic computing, as a new type of computer system, has the advantages of a low power consumption, a high efficiency, and a high fault tolerance. Because it solves the problem of expensive data transfer between the processor and the memory, it has become a hot area worthy of in-depth study [1,2,3,4,5]. As an emerging nonvolatile device, the memristor changes its own resistance through the stimulation of the external voltage, similarly to a biological synapse, simulating the change in synaptic weight in a biological synapse. It is considered an ideal device for studying neuromorphic computing [6,7,8]. The low power consumption, scalability, high density, and nonvolatility of memristors facilitate the realization of synaptic behaviors in biological neurons [9,10,11], which are capable of performing, learning, memory, and other complex neural behaviors [12,13,14,15], making them a key element in neuromorphic computing research. Previous memristor research achieved a variety of synaptic behaviors, such as short-term plasticity, excitatory postsynaptic current (EPSC), paired-pulse facilitation (PPF), long-term plasticity (STP), spike-rate-dependent plasticity (SRDP), and pulse-time-dependent plasticity (STDP) [16,17,18,19,20].

In an effort to find suitable materials for fabricating artificial synapses, a wide variety of materials have come into the field of researchers, such as metal oxide materials [21], inorganic materials [22,23], chalcogenides [24], perovskites [25,26,27,28,29], and 2D materials [30,31]. Most of the materials reported in the literature are complex to prepare and incompatible with organisms [32,33,34], making them difficult to use due to a lack of environmentally friendly disposal methods and for posing potential threats to the environment. Therefore, to produce biocompatible devices at a low cost, new materials must meet these requirements, using readily available natural compounds. Organic materials are widely used in the preparation and research of resistive switching layers due to their low cost and nonpolluting environment [35,36,37]. Memristors made of natural biomaterials are widely used in wearable, compatible, and environmentally friendly smart electronic devices; among these materials are lignin [38], pectin [39], chitosan [40], and anthocyanins [41], which have been used as the memristors’ active layer of a device. In addition, protein materials are also widely used in artificial synapse devices because of their good biocompatibility, degradability, specificity, and ease of processing. By regulating the synthesis process of protein materials or modifying them after synthesis, their structure and properties can be adjusted. As a protein material, egg albumen has good degradability and biocompatibility and can be absorbed by the human body. Moreover, egg albumen is a protein material with variable electrical conductivity, which is similar to the neuroplasticity of biological synapses and can simulate the synaptic connections between biological neurons. Therefore, egg albumen has been used as an active or dielectric layer in fabricating high-performance resistive switching devices [36] and thin-film transistors [34]. However, in-depth research on biomaterial-related synaptic bionics and neuromorphic computing remains incomplete.

Biomemristors have been extensively studied recently. Yan et al. used EA as an active layer to prepare a flexible W/EA/ITO/PET device, which can shift from paired pulse facilitation to paired pulse depression by changing the amplitude of the pulse voltage [42]. Zhou et al. prepared a superflexible protein paper substrate with good physical flexibility and excellent electrical properties, and, through a simple thermochemical reaction, they prepared an Au/EA/Au memristor, which still showed bipolar I-V characteristics in the bending state and type-switching behavior [43]. Sung et al. prepared Al/EA–GQD/ITO biomimetic synapse devices based on a hybrid nanocomposite of egg albumin (EA) and graphene quantum dots (GQDs). The I-V characteristics of the device exhibited clockwise hysteresis behavior under continuous positive and negative voltage scans [44]. Ghosh et al. used aloe vera flower extract as the active layer to create a biomemristor. Their device can achieve a high current-switching ratio and stable cyclability and exhibits two different extraction solutions in the preparation state and in the chemical reduction state [45]. Sun et al. embedded graphene into anthocyanins to make memristors and manipulated the I-V properties through the protonation of graphene to produce a self-selected memristor effect [41]. Saha et al. synthesized water-soluble sodium caseinate (NaCas), using the natural casein extracted from edible animal milk, and used it to prepare an Al/NaCas/ITO biomemristor. The device showed a large switching-current ratio, a long retention time, and a stable cycle durability [46]. It is worth studying the use of biological memristors to further realize artificial synapses and artificial neurons [47,48,49,50].

Polymethyl methacrylate (PMMA) easily forms a film due to its high transmittance and is often used as an insulating layer in organic thin-film transistors. Graphene quantum dots (GQDs), as a new type of quantum dot, have a good biocompatibility, a low cytotoxicity, and photoluminescence properties, making them very suitable as dopants for biological materials. In this paper, Al/PMMA/egg albumen (EA)–GQDs/PMMA/indium tin oxide (ITO) biomemristors were fabricated using the egg protein as a dielectric layer. The PMMA was added as an insulating layer on both sides of the EA dielectric layer, and the GQDs were doped to increase the light-sensitive properties of the device. The electrons in the GQDs were injected from the quantum dots into the dielectric layer or into the adjacent quantum dots under optical excitation; a pathway composed of GQDs was formed in the EA–GQDs dielectric layer, and electrons were transmitted on this pathway. The device successfully performed nine brain synaptic functions: excitatory postsynaptic current (EPSC), paired-pulse facilitation (PPF), short-term potentiation (STP), short-term depression (STD), the transition from short-term plasticity to long-term plasticity, spike-timing-dependent plasticity (STDP), spike-rate-dependent plasticity (SRDP), the process of learning, forgetting, and relearning, and Pavlov associative memory under UV light stimulation. The successful simulation of the synaptic behavior of this device provides the possibility for biomaterials to realize neuromorphic computing.

## 2. Materials and Methods

### 2.1. Device Fabrication

Fresh eggs were cut, and the egg white solution was extracted from them. We mixed 0.5 mL of EA and 5 mL of deionized water at a ratio of 1:10, placed it in an ultrasonic cleaner to disperse for 15 min, and mixed it thoroughly with 2.75 mL of 30% GQDs. The PMMA powder (400 mg) was mixed with 10 mL of anisole solution and stirred with a magnetic stirrer for 12 h. The glass with the ITO bottom electrode was ultrasonically cleaned with absolute ethanol and deionized water for 15 min each. The PMMA solution was dropped onto the ITO-conductive glass and spin-coated at 1000 rpm for 20 s, and the spin-coated PMMA substrate was placed in a drying oven at 100 °C for 1 h. The mixed solution of EA and GQDs was drop-coated onto the PMMA insulating layer and spin-coated at 4000 rpm for 40 s, and the spin-coated substrate was placed in a drying oven at 105 °C for 10 min. The PMMA solution was drop-coated onto the EA layer, spin-coated at 1000 rpm for 20 s, and then placed in a drying oven to cure for 1 h. A layer of Al electrodes was deposited onto the upper PMMA insulating layer using the vacuum evaporation method. Finally, the prepared device was placed in a vacuum-drying oven and dried at 105 °C for 10 min for annealing.

### 2.2. Instrument

The UV–Vis spectra of the SA materials were obtained with an ultraviolet–visible spectrophotometer (UV/VIS, TU-1901) (Beijing Purkinje General Instrument Co., Ltd., Beijing, China). The electrical characteristics of the prepared device were tested using a semiconductor parameter tester (Keithley 4200) (Keithley, Solon, OH, USA). During the electrical test of the device, the ITO bottom electrode was grounded, and a bias voltage was applied to the aluminum top electrode at the same time. The cross sections of the devices were characterized by scanning electron microscopy (Hitachi S-3400 N, Hitachi, Tokyo, Japan).

## 3. Results

**Figure 1:**
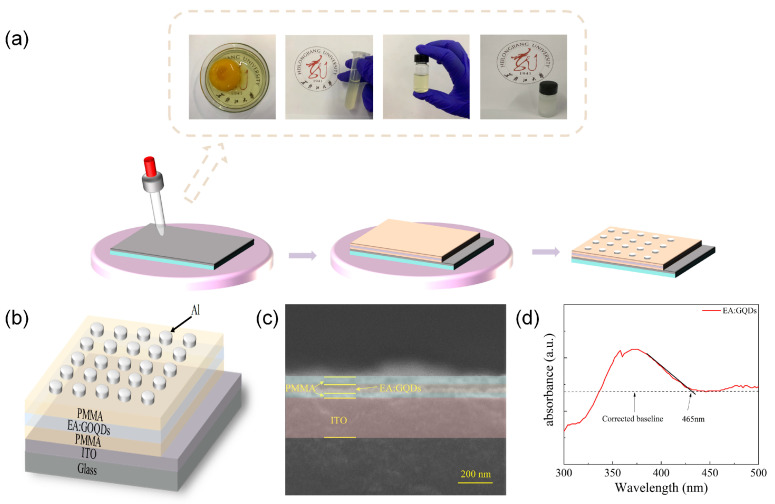
(**a**) Al/PMMA/EA–GQDs/PMMA/ITO’s fabrication flow chart. (**b**) Schematic diagram of the structure of the device. (**c**) SEM photos of the cross-section of the device. (**d**) UV–Vis absorption spectra of the EA–GQD film.

The manufacturing flow chart of the Al/PMMA/EA–GQDs/PMMA/ITO device is shown in Figure 1a. The device’s structure is Al/PMMA/EA–GQDs/PMMA/ITO, as shown in Figure 1b. The top electrode material is aluminum; the active layer is PMMA/EA–GQDs/PMMA, and the bottom electrode is ITO. The cross-section of the device is observed using a scanning electron microscope, as shown in Figure 1c, with PMMA (53 nm), EA (28 nm), PMMA (10 nm), and ITO (200 nm), in order from top to bottom. The optical analysis of the EA–GQD film using the UV–visible light spectrum is shown in Figure 1d. The wavelength corresponding to *E_g_* is obtained through the intersection of the edge tangent line of the absorption peak and the correction baseline. The edge of the absorption peak of the EA–GQD film is located at a wavelength of *λ* = 465 nm, according to the following:

(1) $$E_{g}=\frac{hc}{\lambda }$$

In Equation (2), *E_g_* is the band gap width; *h* = 6.62 × 10^−34^ J·s is Planck’s constant; *c* = 3 × 10^8^ m/s is the photon speed, and *λ* is the absorption wavelength. The bandgap of EA–GQDs is approximately 2.67 eV.

**Figure 2:**
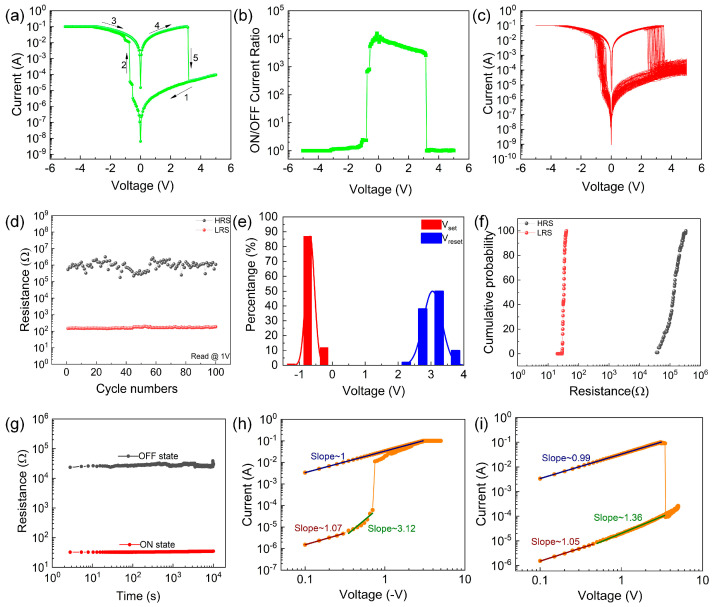
Electrical characteristics of the device. (**a**) Typical I-V characteristics. (**b**) ON/OFF current ratio. (**c**) 100-scan I-V characteristics. (**d**) Switching-endurance property. (**e**) Threshold voltage distribution histogram. (**f**) Resistance accumulation probability. (**g**) Retention property. (**h**) I-V characteristic curve under negative voltage. (**i**) I-V characteristic curve under a positive voltage.

Figure 2a shows the electrical characteristics of the device. In the negative voltage scan of 0–5.00 V, when the set voltage (V_set_) is −0.75 V, the device changes from a high resistance state (HRS) to a low resistance state (LRS), realizing the set process. In the positive voltage scan of 0–5.00 V, the device changes from the LRS to the HRS under the reset voltage (V_reset_) of 3.20 V, completing the reset process. Figure 2b is a schematic diagram of the switching-current ratio of the device. The maximum on/off current ratio of the device is approximately 10^4^. The device can perform 100 cycles in the same cell, as shown in Figure 2c. The results show that the Al/PMMA/EA–GQDs/PMMA/ITO device has a stable resistance-switching memory behavior.

The device proposed in the manuscript can complete 100 cycles, and Figure 2d shows the distribution of the high and low resistance states of the device under the limiting voltage of 1 V during the cycle. Figure 2e shows the threshold voltage distribution of the device. The average value of V_set_ for the Al/PMMA/EA–GQDs/PMMA/ITO device is −0.67 V, with a standard deviation of 0.13 V. The average value for the V_reset_ process is 3.05 V, with a standard deviation of 0.28 V. These results indicate that the device exhibits an excellent stability. Figure 2f shows the cumulative resistance probability of the Al/PMMA/EA–GQDs/PMMA/ITO device for 100 consecutive scans of the same cell at a voltage of 0.1 V. The median values of R_HRS_ and R_LRS_ are 1.85 × 10^5^ Ω and 30.01 Ω, respectively. The R_LRS_ distribution of the equipment is relatively concentrated, with a coefficient of variation of approximately 0.19, and the coefficient of variation of the RHRS of the device is 0.46, indicating the good consistency of the device. The retention time of the device was tested at 1 V, as shown in Figure 2g. The test results show that the device is maintained for more than 1 × 10^4^ s in both the HRS and the LRS: the HRS remains at 2.75 × 10^4^ Ω, and the LRS remains at 34.37 Ω. The fitting results of the I–V characteristic curve of the Al/PMMA/EA–GQDs/PMMA/ITO memristor under positive and negative voltages in double logarithmic coordinates are shown in Figure 2h,i. In Figure 2h, the slope of the fitted curve in the LRS is one, showing linearity, which is consistent with the ohmic conduction mechanism (*I*∝*V*). The fitting curve of the HRS is divided into two parts, and the logarithmic curve of the HRS is a quadratic curve that shows a power relationship (*I*∝*V*^2^). In the low voltage region, the slope of the fitting curve is 1.07, which is consistent with the ohmic conduction mechanism. In the high voltage region, the slope of the fitting curve is 3.12, which is consistent with the space-charge-limited current effect. When the bias voltage reaches V_SET_, the conductive filament connects the upper and lower electrodes, and the logarithmic I-V curve of the LRS becomes linear. The same is true for the positive voltage region, as shown in Figure 2i. Compared to the devices of undoped GQDs, the introduction of GQDs increases the number of defects in the dielectric layer so that more electrons injected into the dielectric layer are captured by the traps.

**Figure 3:**
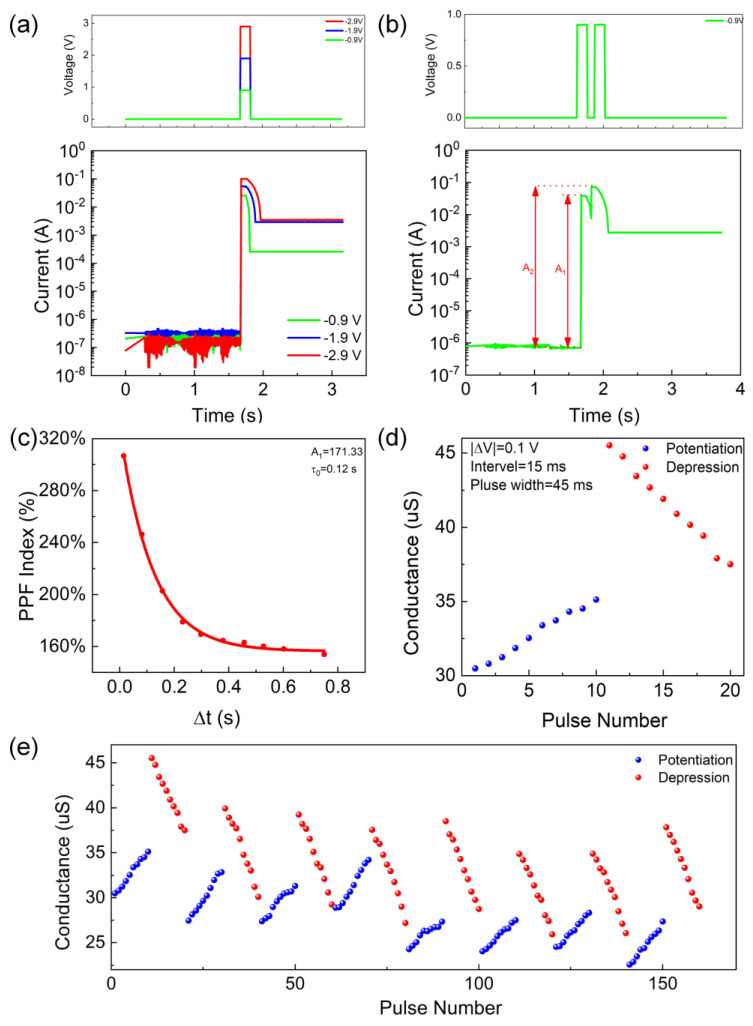
Short-term plasticity of the device: (**a**) EPSC behaviors corresponding to pulse waveforms with different amplitudes. (**b**) PPF neurobehavior. (**c**) The PPF index (A_2_/A_1_) is affected by two consecutive presynaptic pulses, and Δt is the time between two presynaptic pulses. (**d**) Synaptic weight change ΔW(A_2_ − A_1_/A_1_) affected by two consecutive presynaptic pulses. Potentiation and depression behaviors of the device. (**e**) Potentiation and depression behaviors under 100 consecutive pulses.

To simulate the excitatory postsynaptic current (EPSC) behavior of the Al/PMMA/EA–GQDs/PMMA/ITO artificial synapse, the bottom electrode ITO is connected to the ground to simulate the presynaptic membrane, and the top electrode Al is connected to the excitation voltage to simulate the postsynaptic membrane. As shown in Figure 3a, the current increased rapidly with the increasing excitation voltage; after the application of the excitation voltage was stopped, the current gradually decreased and, finally, stabilized. The EPSC current of the device increases with increasing negative excitation voltage under different levels of excitation stimulation, but its degree of increase decreases gradually. The higher the EPSC current, the longer it takes to drop to a stable value; in this scenario, the corresponding stable current is also higher, indicating the excellent nonvolatility of the device. As shown in Figure 3b, the device exhibits paired-pulse facilitation (PPF) behavior in response to an excitation stimulus. The response current for the first stimulus A_1_ is 3.77 × 10^−2^ A, and the response current for the second stimulus A_2_ is 7.17 × 10^−2^ A. The effect of this on the artificial synapse is expressed as the PPF exponent (A_2_/A_1_) for two stimuli with different time intervals (Δt). As shown in Figure 3c, the PPF index gradually decreases from 240% to 160% as the Δt between the two excitations increases. The excitation shown in Figure 3d is applied to the device to simulate the potentiation and depression behaviors. The artificial synapse exhibited an increase in conductance and a decrease in conductance during the potentiation and depression behaviors, respectively. In Figure 3e, the device potentiation and depression behaviors under ten consecutive excitation stimuli, showing that the device has a good synaptic plasticity.

**Figure 4:**
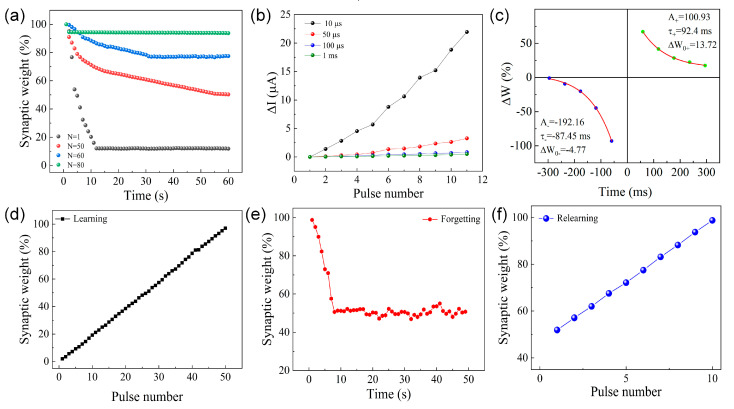
(**a**) Variation in synaptic weights with retention time after the application of different numbers of excitations. (**b**) Spike-rate-dependent plasticity. (**c**) Spike-time-dependent plasticity. (**d**) Learning behavior, (**e**) forgetting behavior, and (**f**) relearning behavior of human synapses.

In the human brain, short-term memory can be transformed into long-term memory by repeated stimuli: this is one of the most important properties of humans’ memory function. The transition from short-term plasticity (STP) to long-term plasticity (LTP) in the Al/PMMA/EA–GQDs/PMMA/ITO memristors was investigated. As the number of excitations increased, the decrease in synaptic weights gradually decreased, and the stable synaptic weights increased when the excitation was removed. In particular, the synaptic weight decreased the fastest when one excitation was applied and the least when eighty consecutive excitations were applied but, finally, returned to a stable state, as shown in Figure 4a. In Figure 4b, the rising trend of the relative current change (A_2_-A_1_) with increasing excitation frequency is gradually significant, demonstrating the good pulse-rate-dependent plasticity behavior of the device. At the same time, as the excitation rate increases, the synaptic weight increases more obviously, and the synaptic weight reaches its maximum when the excitation interval is 10 μs. The pulse-time-dependent plasticity (STDP) observed in biological synapses is generally expressed with the following equation:

(2) $$\Delta W=\left\{\begin{array}{c} A_{+}e^{-\left|\Delta t\right|/t_{+}}+{\Delta W}_{0+},\;\;\Delta t>0\\ A_{-}e^{-\left|\Delta t\right|/t_{-}}+{\Delta W}_{0-},\;\;\Delta t<0 \end{array}\right.$$

where A_+_ and A_−_ represent the scaling factors; τ+ and τ− represent the time constants, and ∆W represents the synaptic weights. The STDP curve of the device fits well with Equation (2), and the relative change in synaptic weight (ΔW) decreases as |Δt| increases, as shown in Figure 4c. To implement the learning experience, the Al/PMMA/EA–GQDs/PMMA/ITO synaptic device was used to simulate the learning, forgetting, and relearning process. First, it can be seen from Figure 4d that, after 50 consecutive excitation stimuli, the synaptic weight gradually increases with the increasing stimulation, which represents the learning behavior of the synapse. Then, after removing the stimulus, it is observed that the synaptic weights decay spontaneously with time and stabilize after a period of time, a process which represents the forgetting behavior of the synapses (Figure 4e). This is similar to the phenomenon where the information learned by a human brain is often partially forgotten after a period of time. Finally, after the spontaneous decay, 10 excitations were sufficient for the synaptic device to acquire the same level of weights as in Figure 4d (Figure 4f). The artificial synaptic device relearns previously forgotten information faster than the first time, a phenomenon similar to the relearning behavior in the brain.

When a negative voltage is applied to the Al electrode, the electrons tunnel directly into the EA–GQDs layer. As the voltage gradually increases, the energy band gradually bends so that a large potential barrier exists between the PMMA and EA–GQD layers, hindering the formation of internal conductive filaments. When the voltage increases to V_set_, the trap state of the active layer is filled, and a conductive path is gradually formed, causing the resistance state of the device to suddenly change from a high resistance state (HRS) to a low resistance state (LRS). At this point, the electrons are allowed to travel freely in the device, and these electrons are continuously released from their trapped state. Therefore, the conduction mechanism of the LRS can be explained with ohmic conduction. When the trap states of the active layer are filled with the injected electrons, the device remains in the LRS even when gradually decreasing negative voltages are applied. In contrast, when a positive voltage is applied to the Al electrode, the electrons in the dielectric layer are continuously released from the trap center. When the voltage increases to V_reset_, the conductive path is destroyed, causing the device to switch from the LRS to the HRS.

**Figure 5:**
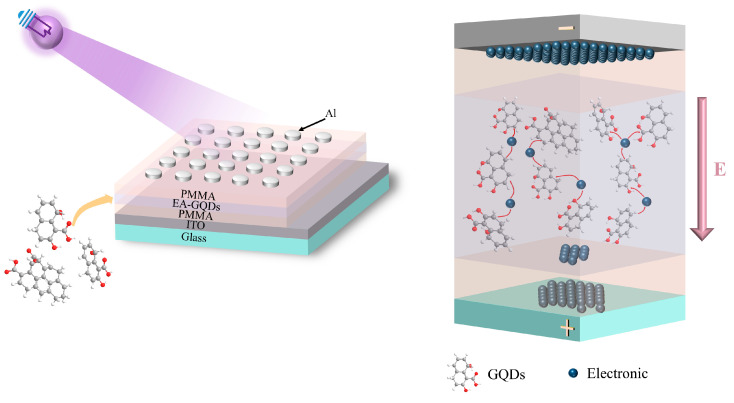
Conduction mechanism of the Al/PMMA/EA–GQDs/PMMA/ITO device under UV irradiation.

A UV light with a wavelength of 395 nm and a power of 171.42 mW/cm^2^ was applied to the Al/PMMA/EA—GQDs/PMMA/ITO memristor. Figure 5 shows the conduction mechanism of the device under UV irradiation. When a negative voltage is applied to the Al electrode, since the PMMA layer is an insulating layer, a large number of positive and negative charges accumulate on both sides, and a top-to-bottom electric field is generated in the EA dielectric layer. Under ultraviolet irradiation, the electrons in GQDs may be injected from the quantum dots into the dielectric layer or into the adjacent quantum dots under photoexcitation. When the concentration of doped quantum dots is high, a pathway composed of GQDs is formed in the EA–GQD dielectric layer, and electrons are transported along this pathway under optical excitation. The generation of vias provides more electron transport paths, enhances the charge-injection efficiency, and, thus, increases the conductivity of the dielectric layer. The different electrical properties of the Al/PMMA/EA–GQDs/PMMA/ITO memristor with or without UV irradiation were analyzed. In this analysis, the pulsed electrical signal was used as a bell signal, and the ultraviolet irradiation was used as a food signal. The electrical properties of the device under these two signals was used as a stimulus response to realize Pavlovian associative memory.

**Figure 6:**
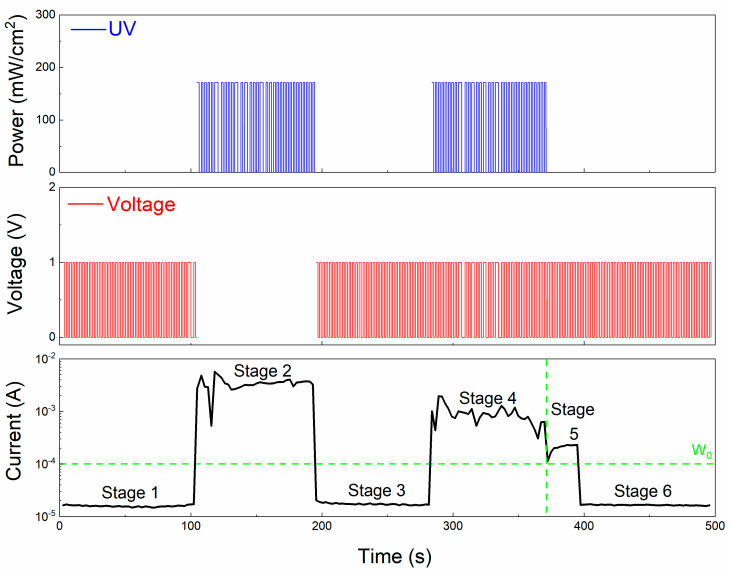
Pavlovian associative memory of the Al/PMMA/EA–GQDs/PMMA/ITO device under UV irradiation and pulsed stimulation.

Applying voltage to the Al/PMMA/EA–GQDS/PMMA/ITO memristor is a ringtone stimulus, and applying UV irradiation to the memristor is a food stimulus, as shown in Figure 6. In the figure, W_0_ is 1 × 10^−4^ A. When the synaptic weight is greater than W_0_, the dog secretes saliva, and, when the synaptic weight is less than W_0_, the dog does not secrete saliva. When the ringtone stimulus was applied, the current of the device was 2 × 10^−5^ A; the synaptic weight was less than the weight W_0_ of salivation, and the dog did not salivate. When the food stimulus was applied, the current of the device was 3 × 10^−4^ A; the weight was greater than the weight W_0_ of salivation, and the dog would salivate. The Al/PMMA/EA–GQDs/PMMA/ITO memristor realizes Pavlovian associative memory under the continuous action of pulsed voltage stimulation and UV irradiation stimulation. Figure 6 is divided into six stages. Stage 1 consists of the bell stimulus; the synaptic weight is smaller than the salivation weight W_0_; the dog does not secrete saliva. Stage 2 covers the food stimulus; the synaptic weight is greater than the saliva secretion weight W_0_; the dog secretes saliva. Stage 4 comprises food. The stimulation of the food and the bell stimulation work together, and the dog secretes saliva. In stage 5, after the food stimulation and bell stimulation, the dog is immediately stimulated by the bell. At this time, the synaptic weight decreases, but it is still greater than the salivation weight W_0_, and the dog secretes saliva. This process corresponds to the associative memory stage. If the dog continues to be stimulated by the bell, the synaptic weight will continue to decrease, which will lead to it weighting less than the weight of the saliva secretion W_0_. In this case, the dog would not secrete saliva, meaning that this process corresponds to the forgetting stage.

## 4. Conclusions

In this paper, Al/PMMA/EA–GQDs/PMMA/ITO biomemristors were fabricated using egg protein as a dielectric layer. The PMMA was added to the upper and lower sides of the EA dielectric layer as an insulating layer, and the GQDs were doped in the EA resistive layer to increase the light-sensitive properties of the device. The electrons in the GQDs were injected from the quantum dots into the dielectric layer or into the adjacent quantum dots under the excitation of light. The EA–GQDs dielectric layer formed a pathway composed of GQDs, and electrons were transported on this pathway. After testing, it could be seen that the device has good electrical characteristics. The device successfully simulates the following synaptic functions: EPSC, PPF, STP, STD, the transformation of short-term plasticity to long-term plasticity, STDP, SRDP, the process of learning, forgetting, and relearning, and Pavlov associative memory under UV light stimulation. The results show that the Al/PMMA/EA–GQDs/PMMA/ITO memristor has a strong potential for simulating biological synapses, which is of great significance to the development of neuromorphic computing systems.

## Data Availability

The data sets used and/or analyzed during the current study are available from the corresponding author upon reasonable request.
